# Effects of Insulin Therapy on Myocardial Lipid Content and Cardiac Geometry in Patients with Type-2 Diabetes Mellitus

**DOI:** 10.1371/journal.pone.0050077

**Published:** 2012-12-03

**Authors:** Drazenka Jankovic, Yvonne Winhofer, Miriam Promintzer-Schifferl, Evelyne Wohlschläger-Krenn, Christian Heinz Anderwald, Peter Wolf, Thomas Scherer, Gert Reiter, Siegfried Trattnig, Anton Luger, Michael Krebs, Martin Krssak

**Affiliations:** 1 Division of Endocrinology and Metabolism, Department of Internal Medicine III, Medical University of Vienna, Vienna, Austria; 2 Metabolic Unit, ISIB CNR, Padova, Italy; 3 Agathenhof, Specialized Hospital for Metabolic Diseases, Micheldorf, Austria; 4 Siemens Healthcare, Graz, Austria; 5 Department of Radiodiagnostics, Centre of Excellence High-Field MR, Medical University of Vienna, Vienna, Austria; Clermont Université, France

## Abstract

**Aims/Hypothesis:**

Recent evidence suggests a link between myocardial steatosis and diabetic cardiomyopathy. Insulin, as a lipogenic and growth-promoting hormone, might stimulate intramyocardial lipid (MYCL) deposition and hypertrophy. Therefore, the aim of the present study was to investigate the short-term effects of insulin therapy (IT) on myocardial lipid content and morphology in patients with T2DM.

**Methods:**

Eighteen patients with T2DM were recruited (age 56±2 years; HbA1c: 10.5±0.4%). In 10 patients with insufficient glucose control under oral medication IT was initiated due to secondary failure of oral glucose lowering therapy (IT-group), while 8 individuals did not require additional insulin substitution (OT-group). In order to assess MYCL and intrahepatic lipid (IHLC) content as well as cardiac geometry and function magnetic resonance spectroscopy (MRS) and imaging (MRI) examinations were performed at baseline (IT and OT) and 10 days after initiation of IT. Follow up measurements took place 181±49 days after IT.

**Results:**

Interestingly, basal MYCLs were 50% lower in IT- compared to OT-group (0.41±0.12 vs. 0.80±0.11% of water signal; p = 0.034). After 10 days of IT, an acute 80%-rise in MYCL (p = 0.008) was observed, while IHLC did not change. Likewise, myocardial mass (+13%; p = 0.004), wall thickness in end-diastole (+13%; p = 0.030) and concentricity, an index of cardiac remodeling, increased (+28%; p = 0.026). In the long-term MYCL returned to baseline, while IHCL significantly decreased (−31%; p = 0.000). No acute changes in systolic left ventricular function were observed.

**Conclusions/Interpretation:**

The initiation of IT in patients with T2DM was followed by an acute rise in MYCL concentration and myocardial mass.

## Introduction

The pathogenesis of diabetic heart disease is multi-factorial and complex. Putative mechanisms include metabolic disturbances, myocardial fibrosis and small vessel disease [1]. High dietary intake of free fatty acids may result in intracellular accumulation of potentially toxic intermediates of the lipid metabolism, all of which lead to impaired myocardial performance and morphological changes [2], [3]. At the late stage of the disease myocyte loss and replacement fibrosis is increased, indicating cardiac remodeling in patients with type-2 diabetes mellitus (T2DM) [4], [5]. In accordance, assessment of cardiac lipid metabolism by means of magnetic resonance spectroscopy in obese patients with T2DM and non-ischemic cardiomyopathy demonstrated increased intramyocardial lipid content (MYCL) [6]–[8]. However, to date contradictive results have been published concerning the short term effects of MYCL accumulation (steatosis) on cardiac function [7], [9].

Growing evidence indicates a potential relationship between chronic hyperinsulinemia in pre-diabetic patients and structural changes of the heart leading to myocardial fibrosis [10], [11]. A crucial role in the pathogenesis of myocardial hypertrophy has been identified for insulin-related cell signaling pathways including the insulin/PI3k/PKB/Akt axis [12]. Consistently, it was demonstrated that disturbances of this pathway induce a decrease in glucose uptake and glucose oxidation and an increase in fatty acid utilization [13].

In a recent study we observed that insulin acutely increases MYCL content and alters cardiac function in the presence of standardized hyperglycemia/hyperinsulinemia (clamp test) in healthy subjects [14]. Therefore we hypothesized that exogenous insulin supply promotes the development of myocardial steatosis and modifies left ventricular contractility in patients with T2DM.

## Methods

### Ethics Statement

The study was approved by the institutional medical ethical committee (Ethics Committee of the Medical University of Vienna) and written informed consent was obtained from all participants. All clinical investigations have been conducted according to the principles expressed in the Declaration of Helsinki.

### Study Participants

Eighteen patients with T2DM were recruited from the outpatient service of our department (**Table 1**). Inclusion criteria were insufficient metabolic control under oral anti-diabetic medication at the time point of clinical assessment (HbA1c >8%) and resting blood pressure <150/85 mmHg with or without antihypertensive medication. Patients with previous myocardial infarction, coronary artery disease and/or history of congestive heart failure were excluded. In addition, subjects, who received digitalis and/or thioazolidinediones did not participate, since thiazolidinediones have been shown to affect myocardial lipid content [15]. None of the study participants had been treated with insulin before or presented with type 1 diabetes-related antibodies. All female patients were postmenopausal. Oral anti-diabetic agents prior the initiation of IT included: metformin (n = 13), sulfonylurea or glinide (n = 8), and gliptine (n = 5). Eleven study participants were on lipid lowering therapy with statins and 1 was treated with ezetimibe. Eight patients reported regular intake of combined anti-hypertensive therapy (angiotensin 2 receptor antagonist or ACE inhibitor: n = 3; selective β-blocker: n = 1; calcium channel blocker: n = 1; diuretics: n = 1).

**Table 1:** Clinical and biochemical characteristics of patients with T2DM treated with standardized IT compared with individuals under oral anti-diabetic therapy (OT).

Characteristics	Baseline N = 8 (oral medication, OT) (mean ± SEM)	Baseline N = 10 (standardized IT) (mean ± SEM)	Day 10 N = 10 (standardized IT) (mean ± SEM)	Follow up N = 7 (standardized IT) (mean ± SEM)
Age (years)	53±2	58±3	–	–
Sex (f/m)	4/4	4/6	–	–
Diabetes duration (years)	3±1	9±2‡	–	–
HbA1c (%)	9.8±0.7	11.1±0.4	n. a.	8.3±0.4†
BMI (kg/m^2^)	30±1.7	30±1.7	30±1.7	30±2.3
BP syst./diast. (mmHg)	137±9/77±3	134±4/75±4	122±2/71±5	138±3/81±4
Plasma glucose (mg/dl)	182±14	231±19	150±8*	146±25
Cholesterol (mg/dl)	217±22	195±9	154±8*	163±15†
LDL cholesterol (mg/dl)	135±19	117±10	n. a.	88±13
Triglycerides (mg/dl)	225±64	192±30	166±16	147±41
HDL- cholesterol (mg/dl)	50±5	40±3	n. a.	45±4
Albumin (g/l)	43±1.6	44±1.0	n. a.	44±0.7
Creatinine (mg/dl)	0.84±0.03	0.94±0.11	n. a.	0.89±0.11
Albumin-Crea-Quot (mg/dl)	14±2.4	31±10.4	n. a.	19±7.3
Pro BNP (pg/ml)	38±9	124±66	n. a.	81±42
Insulin dose (IU/day)	n. a.	0±0	39±7*	49±10†

Values are mean ± SEM.

n. a., not assessed.

* p<0.05 baseline IT vs. 10th day of IT,

† p<0.05 baseline IT vs. follow up IT,

‡ p<0.05 baseline oral medication vs. baseline standardized IT.

### Study Design

The time span between the clinical assessment in the outpatient unit and the hospitalization was 7.5±1.8 days (run-in phase). During this time period compliance with the oral medication was strongly recommended and patients were instructed that in case of persistently insufficient metabolic control the initiation of IT was intended. Additionally, patients provided standardized records of self-blood glucose monitoring until the baseline ^1^H-magnetic resonance imaging (-MRI) and spectroscopy (-MRS) examination. Thereafter, standardized IT (three times daily dual release human insulin analogue suspension containing 30% soluble insulin aspart and 70% insulin aspart protamine crystals, NovoMix 30**®** ^,^ Novo Nordisk, Vienna, Austria) was initiated in patients with secondary failure to oral anti-diabetic treatment (mean plasma glucose >190 mg/dl; IT-group). In these patients all blood-glucose lowering agents except for metformin were discontinued simultaneously with the initiation of IT. Likely, due to the improved compliance to the oral-medication during the run-in phase glycemic control ameliorated in 8 out of 18 patients (OT-group, mean plasma glucose <190 mg/dl). Thus, these patients did not require standardized IT corresponding to the study protocol and only baseline MR examinations were performed in this subgroup of patients. During the course of the study no adjustments of lipid lowering and/or anti-hypertensive medication were performed. During the first 10 days of the inpatient setting standardized frequent measurements of blood glucose concentrations (0 h, 3 h, 6 h, 9 h, 12 h, 15 h, 18 h, 21 h; Accu-Check Go Blood Glucose Monitor, Roche Diagnostics, Vienna, Austria) allowed to quickly titrate insulin doses and achieve pre-prandial glucose concentrations of 100–120 mg/dl. Insulin doses were adjusted twice daily by experienced physicians. In addition a standardized diet with 1400 kilocalories per day (fat/carbohydrate/protein: 32%/48%/20%) was administered during inpatient treatment. All patients were advised to adhere to the diet plan after the discharge. Furthermore, structured inpatient diabetes training included recommendations for regular moderate physical activity. Ten days after the initiation of IT MRI and MRS studies were repeated. Patients were discharged on day 10. Clinical and MR follow up examinations were performed in 7 patients 181±49 days after initiation of IT.

### Magnetic Resonance Imaging (MRI) and Spectroscopy (MRS)

All magnetic resonance measurements were performed on a 3.0-T Tim Trio System (Siemens Helathcare, Erlangen, Germany) operated with the Syngo VB15 and VB17 user interface.

#### ^1^H-MRI for myocardial function

Visualization of cardiac function was performed employing retrospective ECG-gated cine true fast imaging with steady-state precession (TrueFISP) sequences in 2-chamber, 4-chamber and short axes orientation. Short axes images were used to quantify left ventricular global function (end-diastolic and end-systolic volume, stroke volume, ejection fraction and myocardial mass) after manual demarcation of endo- and epicardial borders in end-systolic and end-diastolic phase via ARGUS software (Siemens Healthcare, Erlangen, Germany). Papillary muscles and trabecles were counted to the lumen of the left ventricle. Myocardial mass was determined as mean of end-diastolic and end-systolic muscle volume multiplied with a density of 1.05 g/cm^3^. Mid-ventricular short axis slices were analyzed for the assessment of left ventricular wall thickness [16]. All data were normalized to body surface area (BSA) using the Dubois formula (BSA = 0.007184×height^0.725^×weight^0.425^) [17].

Calculation of concentricity ( = left ventricular mass to end-diastolic volume ratio) [18] was performed to evaluate effects on cardiac remodeling. Additionally, a FLASH-based (fast low angle shot) retrospective ECG-gated cine phase contrast sequence was used to determine E/A ratio of mitral inflow (evaluated via ARGUS software) as a measure of left ventricular diastolic function [19], [20].

#### ^1^H-MRS for quantification of myocardial lipid content (MYCL)

Myocardial lipid measurements were performed using localized ^1^H-MRS based on recently introduced methods [7], [14], [20], [21]. Anatomic imaging was used to guide water suppressed Point RESolved Spectroscopy (PRESS) sequence (echo time, TE = 30 ms, NS = 16). The volume of the interest (VOI; approx. 6–8 cm^3^) was placed over the interventricular septum. The acquisition of MR signal was performed during multiple breath holds using the multichannel cardiac reception coil provided by the system manufacturer (Siemens Healthcare, Erlangen, Germany) and triggered by ECG signal. For the signal acquisition in the mid-diastole trigger delay was adjusted individually. Repetition time of the sequence was given by the heart beat frequency of individual volunteer. An additional spectrum without water suppression (NS = 8) was used as the internal concentration reference. The spectra were processed by the Spectroscopy Processing tool within Syngo VB15 and VB17 user interface provided by the system manufacturer (Siemens Healthcare, Erlangen, Germany). Careful placement of the VOI, appropriate trigger adjustment and the fact that the spin-echo based sequence suppresses signal from flowing liquids makes the possible contribution of ventricular blood to the tissue water signal negligible.

Water signal intensity was quantified from the spectra without water suppression and individual spectral lines intensities of methyl- [CH_3_-; 0.8–0.9 ppm] and methylene- [-(CH_2_)_n_-; 1.25 ppm] groups of fatty acid chains were quantified from the water suppressed spectra. The myocardial lipid content was than calculated as a ratio of the sum of intensities of methyl- and methylene- group resonances to the intensity of the water resonance. Intensities of lipid and water resonance lines were corrected for the spin-lattice (T1) and spin-spin (T2) relaxation using individual repetition time and already published T1 and T2 relaxation times of skeletal muscle at 3T [22]. The reproducibility of the MRS method used here was tested in our previous paper [14] with the resulting coefficient of variance (CV) for test-retest measurement of 23%, which is a substantial improvement over the CV of 30–39% as reported earlier [20], [21], [23].

#### ^1^H-MRS for the quantification of intrahepatic lipid content (IHLC)

The hepatocellular lipids were measured applying similar short echo time ^1^H-MRS single voxel technique using volume of interest of 3×3×3 cm^3^ placed in the lateral aspect of the liver. Single breath hold protocol without spectral water suppression and twelve acquisitions (NS = 4) with the repetition time of 2 sec were used. Flexible body array coil provided by system manufacturer were used for signal reception. The intrahepatic lipid content were calculated as a ratio of the sum of intensities of -(CH_2_)_n_- (1.25 ppm) and CH_3_- (0.8–0.9 ppm) group resonances to the intensity whole MRS signal including the water resonance. Intensities of lipid and water resonance lines were corrected for the spin-lattice (T1) and spin-spin (T2) relaxation already published T1 and T2 relaxation times at 3T [24].

### Analysis of Plasma Metabolites

All laboratory parameters were measured by routine lab methods (http://www.kimcl.at/).

### Statistical Analysis

All data are presented as mean ± standard error of the mean (SEM). The effect of the insulin treatment on heart function and metabolic parameters was determined by the two-sided, paired Student’s *t* test. Differences at *p*<0.05 were considered significant. Pearson and Spearman analyses were performed to disclose correlation between variables as appropriate (SPSS 18.0 for Mac, Chicago, IL, USA).

## Results

### Clinical Parameters

The mean age was 56±2 years and the mean MYCL content was 0.58±0.09% of water signal. Interestingly, when IT was compared to OT, significantly lower MYCL content (0.41±0.12 vs. 0.80±0.11% of water signal; p = 0.034) and longer diabetes diagnosis duration (9±2 vs. 3±1 years; p = 0.015) was revealed in the IT-group**.** There were no significant differences in blood glucose levels, HbA1c, and lipid profiles at the baseline between the IT- and the OT-group **(** **Table 1** **)**.

**Figure 1:**
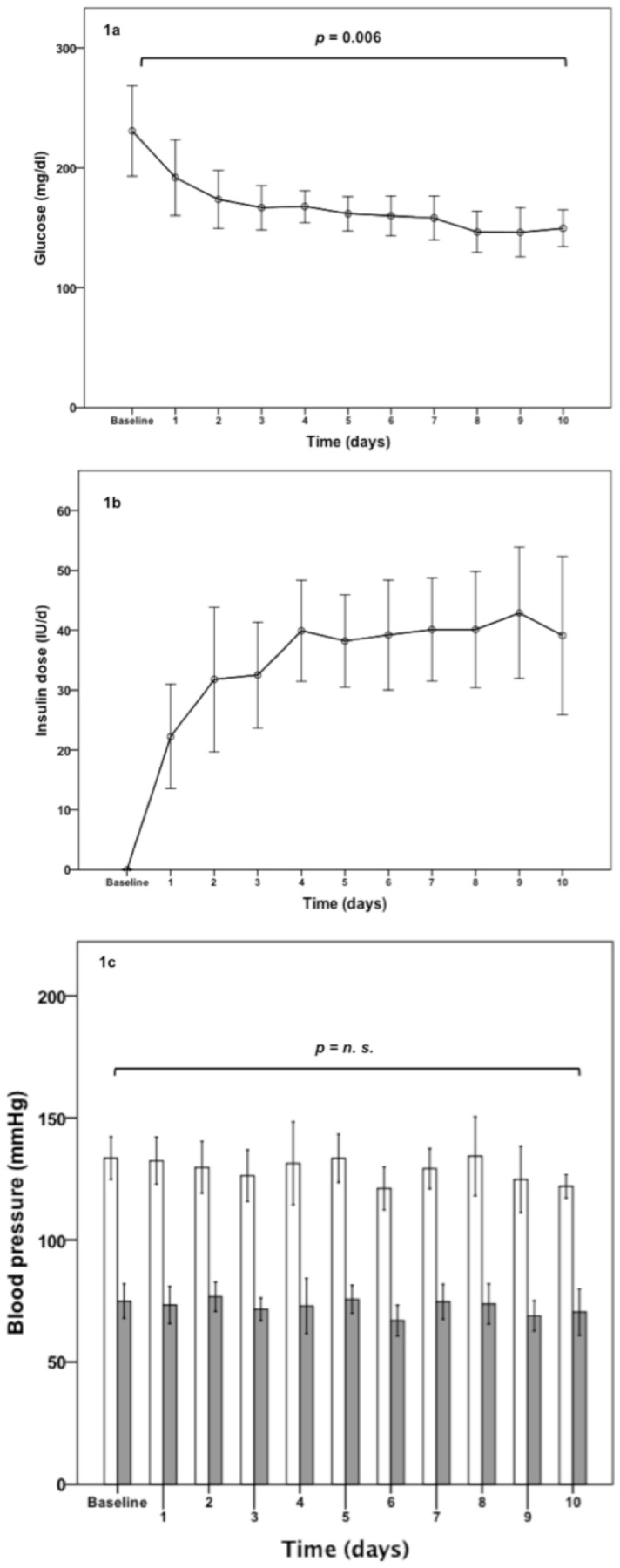
Mean blood glucose concentrations (mg/dl) (a), daily insulin dose (international unit [IU]) (b), systolic and diastolic blood pressure (mmHg) (c) at baseline and during the first 10 days of IT. Empty bars indicate systolic and gray bars diastolic blood pressure values; error bars delineate SEM.

Mean blood glucose concentrations significantly decreased during IT **(** **Table 1** **;** **Figure 1a** **)**. Although lipid-lowering therapy was not modified, serum cholesterol concentrations diminished after short term of IT and did not rebound at follow up **(** **Table 1** **).** **Figure 1** presents the time course of daily insulin doses **(b)** as well as systolic and diastolic blood pressure **(c)** during the inpatient treatment. At follow up (181±49 days after the initiation of IT) a reduction in HbA1c (8.3±0.4%; p = 0.004) documented improved metabolic control **(** **Table 2** **)**. Moreover, a positive correlation was found between albumin-creatinine-quotient and the following clinical features: duration of the disease (Pearsońs r = 0.59; p = 0.012), plasma glucose (Pearsońs r = 0.74; p = 0.001) and HbA1c levels (Pearsońs r = 0.51; p = 0.036) as well as MYCL concentration at day 1 (Pearsońs r = 0.52; p = 0.029)**.**

**Table 2:** Results of MR imaging studies.

Left ventricular variable	Baseline N = 8 (oral medication, OT) (mean ± SEM)	Baseline N = 10 (standardized IT) (mean ± SEM)	Day 10 N = 10 (standardized IT) (mean ± SEM)	Follow up N = 7 (standardized IT) (mean ± SEM)
Heart rate (min^1^)	75±2.8	76±4.1	73±4.6	72±3.1
Ejection fraction (%)	71±2.8	71±2.5	73±3.3	73±3.5
End-diastolic volume (ml/m^2^)	59±3.5	55±5.0	49±4.8	52±5.3
End-systolic volume(ml/m^2^)	18±1.9	17±2.5	14±2.5	14±2.4
Stroke volume(ml/m^2^)	42±2.6	38±2.8	36±3.4	38±4.3
Cardiac index(l/min/m^2^)	3.1±0.2	2.9±0.2	2.6±0.3	2.7±0.2
Myocardial mass(g/m^2^)	58±3.9	54±4.0	62±4.3*	62±4.5†
Thickness in ED (mm/m^2^)	8.1±0.7	8.6±0.5	9.7±0.6*	9.5±0.6
Concentricity (g/ml)	0.88±0.08	0.96±0.08	1.23±0.11*	1.15±0.12
E/A ratio	1.06±0.11	0.93±0.05	0.92±0.06	0.87±0.09

Values are mean±SEM.

ED, end-diastole.

* p<0.05 baseline IT vs. 10th day of IT,

† p<0.05 baseline IT vs. follow up IT.

### Cardiac Function and Morphology

Ten days after the initiation of IT alterations in myocardial mass (+13%) and wall thickness at the end-diastole (+13%) were observed **(** **Table 2** **)**. Moreover, cardiac remodeling, displayed by concentricity, emerged after the initiation of IT **(** **Table 2** **)**. However, left ventricular systolic function did not change during the study course **(** **Table 2** **)**. In 12 patients E/A ratio was below 1 indicating diastolic dysfunction, which remained stable under IT. The rise in myocardial mass persisted throughout the follow up period **(** **Table 2** **)**.

### Cardiac and Hepatic Lipid Content during and after IT

**Figure 2:**
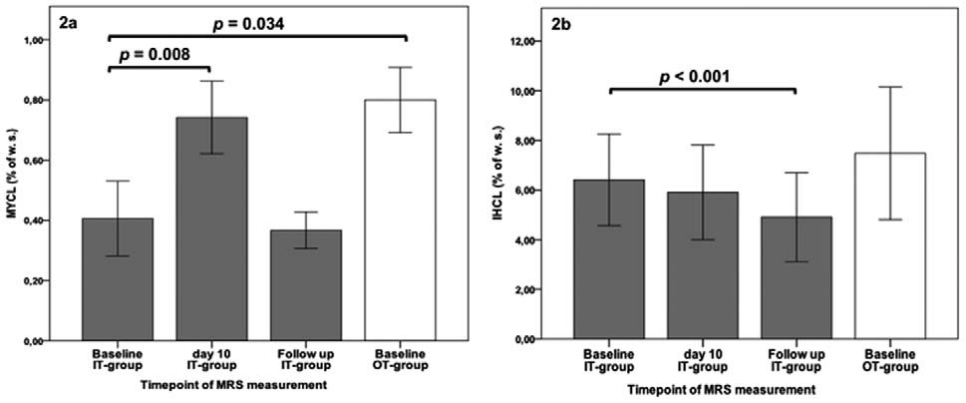
Intramyocardial lipid- (MYCL, given in % of water signal [w. s.]) (a) and intrahepatic lipid concentration (IHCL, given in % of water signal [w. s.]) at baseline, day 10 of IT and during follow up (181±49 days) (b). Gray bars indicate IT-group and empty bar the OT-group; error bars delineate SEM.

**Figure 3:**
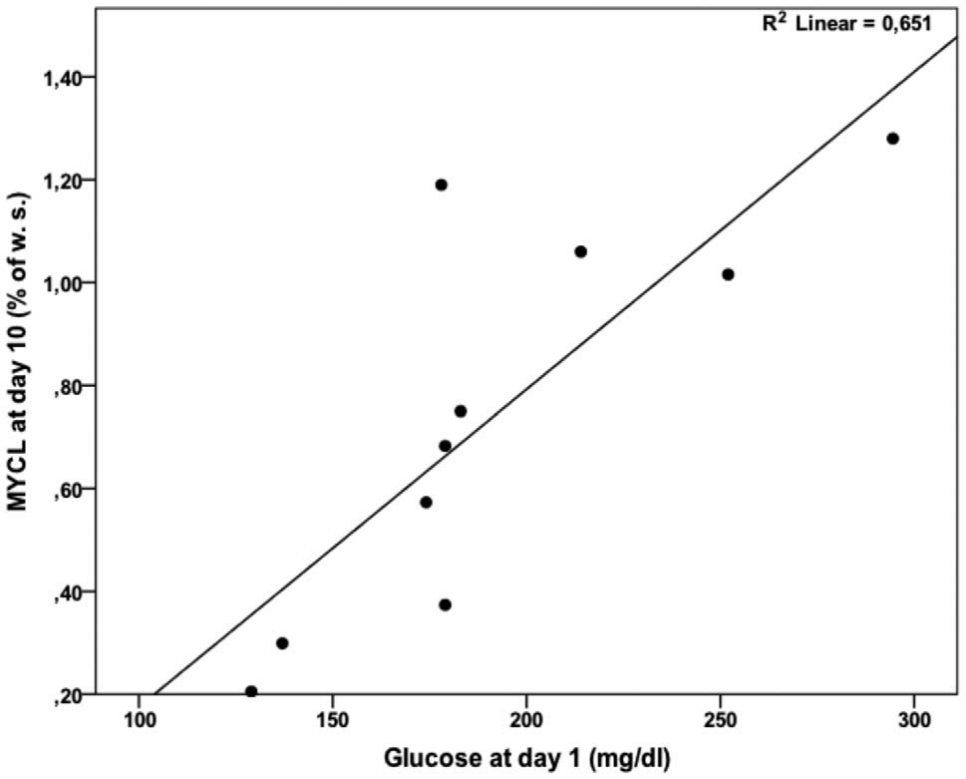
Association between mean glucose concentrations at day 1 and MYCL content at day 10 of IT.

After 10 days of IT MYCL content increased by 80% (p = 0.008; **Figure 2a**), while IHCL tended to decrease, but did not change significantly (p = 0.132; **Figure 2b**). In addition, mean blood glucose concentrations on day 1 were closely associated with MYCL content on day 10 (Pearsońs r = 0.80; p = 0.005; **Figure 3**). Moreover, 181±49 days after IT MYCL returned to baseline (0.37±0.06% of water signal; p = 0.692; **Figure 2a**), whereas IHLC decreased by 31% (5.55±1.93% of water signal; p = 0.000; **Figure 2b**).

## Discussion

The present study shows that the initiation of IT in patients with long standing T2DM and bad metabolic control due to secondary failure of oral glucose lowering therapy is associated with an acute but transient rise in MYCL content and myocardial wall thickness. Furthermore, the observed changes were initially linked to myocardial hypertrophy with preservation of cardiac function.

We have previously shown that insulin infusion designed to achieve near normoglycemia in patients with T2DM augments ectopic lipid accumulation in skeletal muscle and liver [25], [26]. Studies in animal models of T2DM have shown that derangements of myocardial substrate metabolism induce cardiac dysfunction and heart failure. Especially, excessive fatty acid uptake, oxidation and/or storage are considered to be substantially involved in the pathogenesis of diabetic cardiomyopathy [27]–[29]. Moreover, studies in humans illustrate that the myocardial triacylglycerol pool is highly dynamic [14], [30]–[32], significantly contributes to mitochondrial oxidation [33] and thus represents an important biomarker for underlying defects in metabolism [34]. Up to date contradictive results exist concerning the potential direct effects of myocardial steatosis on cardiac function in humans. McGavock at al. has not observed a correlation between myocardial steatosis and left ventricular function in type 2 diabetic patients [9]. In contrast, a study performed in individuals with uncomplicated T2DM indicates that MYCL content is an indirect predictor of myocardial dysfunction [7].

Previous cross-sectional investigations applying cardiac MRS [7], [9], [35] or histology [6] have confirmed elevated MYCLs in metabolic diseases including T2DM, obesity and impaired glucose tolerance. In order to elucidate the underlying mechanisms causative for the development of myocardial steatosis we have recently performed a standardized hyperglycemic clamp test in healthy subjects. We could demonstrate that endogenous hyperinsulinemia in response to hyperglycemia induces an acute increase in MYCL content. [14]. In the present study, we observed a strong correlation between glucose concentrations at day 1 and MYCL at day 10 of IT. Insulin forcefully stimulates myocardial glucose uptake via increased GLUT 4 translocation to the cellular membrane fostering substrate competition between fatty acids and glucose [36], [37]. The resulting switch in mitochondrial substrate utilization from fatty acid- to glucose utilization is mediated mainly by malonyl-CoA. Malonyl-CoA is generated by acetyl-CoA carboxylase (ACC 2) and inhibits CPT I (carnitine palmitoyltransferase) [36], which controls the rate limiting step of mitochondrial FFA-uptake and in turn –oxidation. Insulin also exerts a direct stimulatory effect on ACC, thereby potently suppressing mitochondrial lipid oxidation in the presence of hyperglycemia [38]. In addition increased insulin-mediated uptake of circulating FFA and stimulation of intracellular triglyceride synthesis likely contributed to myocardial lipid accumulation [26].

Our results confirm myocardial steatosis in the expected range in patients with T2DM (OT-group) [21]. However, in subjects with secondary failure of oral glucose lowering therapy MYCL content was in the normal range at baseline. Relative insulin deficiency due to progressive β-cell dysfunction [39] in the IT-group likely contributed to unexpectedly normal (low) MYCL stores in patients with longstanding T2DM**.**

At follow up improvement of metabolic control might have returned MYCL to baseline. These results are in accordance with previous data showing a parallel decrease in MYCL and HbA1c during treatment with pioglitazone and insulin in patients with T2DM [15].

Insulin therapy did not induce an acute rise in hepatic lipid content in the present study, suggesting that myocardial lipids are more sensitive to insulin compared to hepatic lipids. Since the muscle-type CPT1B is 10–100 fold more sensitive to malonyl-CoA compared to liver-type CPT1A [40] the heart might be especially susceptible to substrate competition between fatty acids and glucose. Therefore, insulin might preferentially induce myocardial steatosis in the presence of hyperglycemia.

In our study myocardial mass and thickness acutely increased in response to IT leading to morphological changes of the left ventricle. In accordance, investigations in animal models have shown that exogenous insulin supply induces myocardial hypertrophy and interstitial fibrosis by activation of key mitogenic signaling pathways including angiotensin, MAPK-ERK1/2 and S6K1 [41]–[43]. However, in the present study metabolic and structural changes of the myocardium due to IT were not associated with altered left ventricular function. This observation might be explained by the finding of Condorelli et al. emphasizing that a mild activation of Akt through PI3k, which is primary induced by ligation of transmembrane receptor (e. g. insulin-like growth factor-1 or insulin receptor), leads to cardiac hypertrophy but is not accompanied by cardiac dysfunction [44].

It is a limitation of the current study that the employed MR methods did not allow discerning the precise alterations in myocardial fuel metabolism. Since biopsies of human myocardium are not feasible in a research setting, investigations on human myocardial metabolism are limited to non-invasive techniques. In addition, we cannot exclude a potential effect of the standardized diet and the continued intake of statins on myocardial lipid content during the in-patient setting. However, withholding these treatment regiments would have been ethically unacceptable.

In order to achieve adequate glycemic control insulin therapy is commonly initiated in patients with longstanding T2DM and relative insulin deficiency. The study protocol resembles standardized therapeutic regiments frequently applied in hospital setting worldwide. Thus, the present study provides a mechanistic concept potentially relevant for numerous patients on insulin therapy. We have shown that hallmark-parameters of diabetic cardiomyopathy, myocardial steatosis and hypertrophy, are acutely affected by IT in the presence of hyperglycemia. However, initiation of IT was not associated with short-term changes in myocardial function. Due to the limited number of patients and the short observation period, we cannot draw definitive conclusions or make recommendations for clinical practice on the basis of the present results. Thus, future prospective trials specifically aiming at the elucidation of insulin effects on myocardial lipid metabolism and function enrolling larger patient populations and longer evaluation periods are warranted.

In conclusion, we clearly demonstrate that the initiation of insulin therapy is associated with an acute, but transient, rise in myocardial lipid content in patients with long standing type 2 diabetes and poor metabolic control. Furthermore, changes in the myocardial mass led to left ventricular hypertrophy with preservation of cardiac function in the short term.

## References

[1] Fang ZY, Prins JB, Marwick TH (2004) Diabetic cardiomyopathy: evidence, mechanisms, and therapeutic implications. Endocr Rev 25: 543–567. 15294881 10.1210/er.2003-0012

[2] Rodrigues B, Cam MC, McNeill JH (1998) Metabolic disturbances in diabetic cardiomyopathy. Mol Cell Biochem 180: 53–57. 9546630

[3] Nakayama H, Morozumi T, Nanto S, Shimonagata T, Ohara T, et al (2001) Abnormal myocardial free fatty acid utilization deteriorates with morphological changes in the hypertensive heart. Jpn Circ J 65: 783–787. 11548876 10.1253/jcj.65.783

[4] Nunoda S, Genda A, Sugihara N, Nakayama A, Mizuno S, et al (1985) Quantitative approach to the histopathology of the biopsied right ventricular myocardium in patients with diabetes mellitus. Heart Vessels 1: 43–47. 4093355 10.1007/BF02066486

[5] Regan TJ, Lyons MM, Ahmed SS, Levinson GE, Oldewurtel HA, et al (1977) Evidence for cardiomyopathy in familial diabetes mellitus. J Clin Invest 60: 884–899. 893679 10.1172/JCI108843 PMC372437

[6] Sharma S, Adrogue JV, Golfman L, Uray I, Lemm J, et al (2004) Intramyocardial lipid accumulation in the failing human heart resembles the lipotoxic rat heart. Faseb J 18: 1692–1700. 15522914 10.1096/fj.04-2263com

[7] Rijzewijk LJ, van der Meer RW, Smit JW, Diamant M, Bax JJ, et al (2008) Myocardial steatosis is an independent predictor of diastolic dysfunction in type 2 diabetes mellitus. J Am Coll Cardiol 52: 1793–1799. 19022158 10.1016/j.jacc.2008.07.062

[8] Szczepaniak LS, Dobbins RL, Metzger GJ, Sartoni-D’Ambrosia G, Arbique D, et al (2003) Myocardial triglycerides and systolic function in humans: in vivo evaluation by localized proton spectroscopy and cardiac imaging. Magn Reson Med 49: 417–423. 12594743 10.1002/mrm.10372

[9] McGavock JM, Lingvay I, Zib I, Tillery T, Salas N, et al (2007) Cardiac steatosis in diabetes mellitus: a 1H-magnetic resonance spectroscopy study. Circulation 116: 1170–1175. 17698735 10.1161/CIRCULATIONAHA.106.645614

[10] Stanley WC, Lopaschuk GD, McCormack JG (1997) Regulation of energy substrate metabolism in the diabetic heart. Cardiovasc Res 34: 25–33. 9217869 10.1016/s0008-6363(97)00047-3

[11] Sharma N, Okere IC, Duda MK, Chess DJ, O’Shea KM, et al (2007) Potential impact of carbohydrate and fat intake on pathological left ventricular hypertrophy. Cardiovasc Res 73: 257–268. 17166490 10.1016/j.cardiores.2006.11.007 PMC2700717

[12] Shiojima I, Walsh K (2006) Regulation of cardiac growth and coronary angiogenesis by the Akt/PKB signaling pathway. Genes Dev 20: 3347–3365. 17182864 10.1101/gad.1492806

[13] Coort SL, Bonen A, van der Vusse GJ, Glatz JF, Luiken JJ (2007) Cardiac substrate uptake and metabolism in obesity and type-2 diabetes: role of sarcolemmal substrate transporters. Mol Cell Biochem 299: 5–18. 16988889 10.1007/s11010-006-9372-7 PMC1915649

[14] Winhofer Y, Krssak M, Jankovic D, Anderwald CH, Reiter G, et al (2012) Short-Term Hyperinsulinemia and Hyperglycemia Increase Myocardial Lipid Content in Normal Subjects. Diabetes 61: 1210–1216. 22396203 10.2337/db11-1275 PMC3331780

[15] Zib I, Jacob AN, Lingvay I, Salinas K, McGavock JM, et al (2007) Effect of pioglitazone therapy on myocardial and hepatic steatosis in insulin-treated patients with type 2 diabetes. J Investig Med 55: 230–236. 10.2310/6650.2007.00003 17850734

[16] Waiter GD, McKiddie FI, Redpath TW, Semple SI, Trent RJ (1999) Determination of normal regional left ventricular function from cine-MR images using a semi-automated edge detection method. Magn Reson Imaging 17: 99–107. 9888403 10.1016/s0730-725x(98)00158-1

[17] Burmeister W, Bingert A (1966) [The body surface formula of DuBois and DuBois as a representative of the body cell mass in men between the ages of 21 and 51 years]. Klin Wochenschr 44: 901–902. 5992540 10.1007/BF01711970

[18] Velagaleti RS, Gona P, Chuang ML, Salton CJ, Fox CS, et al (2010) Relations of insulin resistance and glycemic abnormalities to cardiovascular magnetic resonance measures of cardiac structure and function: the Framingham Heart Study. Circ Cardiovasc Imaging 3: 257–263. 20208015 10.1161/CIRCIMAGING.109.911438 PMC3057083

[19] Gerstein HC, Miller ME, Byington RP, Goff DC Jr, Bigger JT, et al (2008) Effects of intensive glucose lowering in type 2 diabetes. N Engl J Med 358: 2545–2559. 18539917 10.1056/NEJMoa0802743 PMC4551392

[20] van der Meer RW, Doornbos J, Kozerke S, Schar M, Bax JJ, et al (2007) Metabolic imaging of myocardial triglyceride content: reproducibility of 1H MR spectroscopy with respiratory navigator gating in volunteers. Radiology 245: 251–257. 17885193 10.1148/radiol.2451061904

[21] Krssak M, Winhofer Y, Gobl C, Bischof M, Reiter G, et al (2011) Insulin resistance is not associated with myocardial steatosis in women. Diabetologia 54: 1871–1878. 21491158 10.1007/s00125-011-2146-0

[22] Krssak M, Mlynarik V, Meyerspeer M, Moser E, Roden M (2004) 1H NMR relaxation times of skeletal muscle metabolites at 3 T. Magma. 16: 155–159. 10.1007/s10334-003-0029-1 15042412

[23] Reingold JS, McGavock JM, Kaka S, Tillery T, Victor RG, et al (2005) Determination of triglyceride in the human myocardium by magnetic resonance spectroscopy: reproducibility and sensitivity of the method. Am J Physiol Endocrinol Metab 289: E935–939. 15972271 10.1152/ajpendo.00095.2005

[24] Krssak M, Hofer H, Wrba F, Meyerspeer M, Brehm A, et al (2010) Non-invasive assessment of hepatic fat accumulation in chronic hepatitis C by 1H magnetic resonance spectroscopy. Eur J Radiol 64: e60–66. 10.1016/j.ejrad.2009.03.062 19406596

[25] Anderwald C, Bernroider E, Krssak M, Stingl H, Brehm A, et al (2002) Effects of insulin treatment in type 2 diabetic patients on intracellular lipid content in liver and skeletal muscle. Diabetes 51: 3025–3032. 12351443 10.2337/diabetes.51.10.3025

[26] Dyck DJ, Steinberg G, Bonen A (2001) Insulin increases FA uptake and esterification but reduces lipid utilization in isolated contracting muscle. Am J Physiol Endocrinol Metab 281: E600–607. 11500316 10.1152/ajpendo.2001.281.3.E600

[27] Aasum E, Hafstad AD, Severson DL, Larsen TS (2003) Age-dependent changes in metabolism, contractile function, and ischemic sensitivity in hearts from db/db mice. Diabetes 52: 434–441. 12540618 10.2337/diabetes.52.2.434

[28] Zhou YT, Grayburn P, Karim A, Shimabukuro M, Higa M, et al (2000) Lipotoxic heart disease in obese rats: implications for human obesity. Proc Natl Acad Sci U S A 97: 1784–1789. 10677535 10.1073/pnas.97.4.1784 PMC26513

[29] Listenberger LL, Ory DS, Schaffer JE (2001) Palmitate-induced apoptosis can occur through a ceramide-independent pathway. J Biol Chem 276: 14890–14895. 11278654 10.1074/jbc.M010286200

[30] Bilet L, van de Weijer T, Hesselink MK, Glatz JF, Lamb HJ, et al (2011) Exercise-induced modulation of cardiac lipid content in healthy lean young men. Basic Res Cardiol 106: 307–315. 21181177 10.1007/s00395-010-0144-x PMC3032894

[31] Hammer S, van der Meer RW, Lamb HJ, Schar M, de Roos A, et al (2008) Progressive caloric restriction induces dose-dependent changes in myocardial triglyceride content and diastolic function in healthy men. J Clin Endocrinol Metab 93: 497–503. 18029455 10.1210/jc.2007-2015

[32] Hammer S, van der Meer RW, Lamb HJ, de Boer HH, Bax JJ, et al (2008) Short-term flexibility of myocardial triglycerides and diastolic function in patients with type 2 diabetes mellitus. Am J Physiol Endocrinol Metab 295: E714–718. 18628354 10.1152/ajpendo.90413.2008

[33] Bucci M, Borra R, Nagren K, Parkka JP, Del Ry S, et al.. (2011) Trimetazidine Reduces Endogenous Free Fatty Acid Oxidation and Improves Myocardial Efficiency in Obese Humans. Cardiovasc Ther. 10.1111/j.1755-5922.2011.00275.x 21884010

[34] Wende AR, Abel ED (2009) Lipotoxicity in the heart. Biochim Biophys Acta. 10.1016/j.bbalip.2009.09.023 PMC2823976 19818871

[35] Iozzo P, Lautamaki R, Borra R, Lehto HR, Bucci M, et al (2009) Contribution of glucose tolerance and gender to cardiac adiposity. J Clin Endocrinol Metab 94: 4472–4482. 19820028 10.1210/jc.2009-0436

[36] Hue L, Taegtmeyer H (2009) The Randle cycle revisited: a new head for an old hat. Am J Physiol Endocrinol Metab 297: E578–591. 19531645 10.1152/ajpendo.00093.2009 PMC2739696

[37] Randle PJ, Garland PB, Hales CN, Newsholme EA (1963) The glucose fatty-acid cycle. Its role in insulin sensitivity and the metabolic disturbances of diabetes mellitus. Lancet 1: 785–789. 13990765 10.1016/s0140-6736(63)91500-9

[38] Witters LA, Kemp BE (1992) Insulin activation of acetyl-CoA carboxylase accompanied by inhibition of the 5′-AMP-activated protein kinase. J Biol Chem 267: 2864–2867. 1346611

[39] DeFronzo R (1997) Pathogenesis of type 2 diabetes: metabolic and molecular implications for identifying diabetes genes. Diabetes Reviews 5: 177–269.

[40] Zammit VA (1999) Carnitine acyltransferases: functional significance of subcellular distribution and membrane topology. Prog Lipid Res 38: 199–224. 10664793 10.1016/s0163-7827(99)00002-8

[41] Nickenig G, Roling J, Strehlow K, Schnabel P, Bohm M (1998) Insulin induces upregulation of vascular AT1 receptor gene expression by posttranscriptional mechanisms. Circulation 98: 2453–2460. 9832492 10.1161/01.cir.98.22.2453

[42] Velloso LA, Folli F, Sun XJ, White MF, Saad MJ, et al (1996) Cross-talk between the insulin and angiotensin signaling systems. Proc Natl Acad Sci U S A 93: 12490–12495. 8901609 10.1073/pnas.93.22.12490 PMC38019

[43] Samuelsson AM, Bollano E, Mobini R, Larsson BM, Omerovic E, et al (2006) Hyperinsulinemia: effect on cardiac mass/function, angiotensin II receptor expression, and insulin signaling pathways. Am J Physiol Heart Circ Physiol 291: H787–796. 16565309 10.1152/ajpheart.00974.2005

[44] Condorelli G, Drusco A, Stassi G, Bellacosa A, Roncarati R, et al (2002) Akt induces enhanced myocardial contractility and cell size in vivo in transgenic mice. Proc Natl Acad Sci U S A 99: 12333–12338. 12237475 10.1073/pnas.172376399 PMC129445

